# Medicine in China

**Published:** 1859-03

**Authors:** John J. Kerr

**Affiliations:** Canton, China


ART. II.—*Medicine in China.* By JOHN J. KERR, M.D., of Canton, China.

THE history of medicine in China is a subject which opens a wide field for investigation, and upon which comparatively little is known. It is evident that in very remote periods there were persons devoted to the practice of the healing art. History and tradition have handed down the names of many persons who were distinguished for skill and success, and some of them have been enrolled among the gods, and temples erected for their worship.

A succession of changes has occurred in the theory and practice of Chinese medicine similar to those which have marked different eras in the history of our profession in Europe. This will appear from what is said of four distinguished men who, like Hippocrates, Galen, and others, stamped the impress of their genius upon their own and succeeding ages. The first and greatest of these masters was *Cheung Chung King*, who lived in the second century of our era, and was cotemporary with Galen. He wrote an original work on fevers, not a single sentence of which, according to the Imperial College, was taken from any more ancient authority. He was distinguished for his heroic practice, giving medicine by the pound instead of ounce. His system prevailed a thousand years, when the second great author, *Lo Shau Chan*, appeared, the peculiarity of whose practice consisted in using bitter and refrigerating medicines.

In the thirteenth century the third great master, *Li Tung Tan*, introduced the elevating and strengthening plan of treating disease. This system prevailed for two centuries, when it was opposed by *Chau Tan Ki*, the fourth great author, whose practice was directed to attenuating and lowering the powers of nature.

The writings of Chinese authors on the different branches of medicine amount to many thousands of volumes, and some of them are very ancient. Their medical literature is not equaled in extent perhaps by that of any other nation. As a specimen the "Pan Trau," or Chinese Herald, may be named. It is a standard work on Materia Medica and Therapeutics, in forty octavo volumes, and contains a list of 756 other works from which it was compiled. Another work, in forty octavo volumes, is called the "Golden Mirror of Eminent Medical Authors," and was compiled by imperial authority.

There are no medical schools in China for the education of physicians. Those who wish to practice learn from private preceptors, and from books, the therapeutical means in common use. A Royal Medical College is maintained at Peking, the Imperial Capital. At the head of it is a president and two deputies, and it is composed of about one hundred members. It is no part of the object of the college to communicate instruction, but the duty of the members is to attend on the royal family and court, and to superintend the publication of medical books.

The number of physicians in China in proportion to the population is perhaps as great as in any other country, and they are divided, as in Europe and America, into regular and irregular. The study of the books which treat of the established methods of cure entitles a man to the standing which belongs to the regular profession. It is probable, however, that the great mass of those who practice are to be reckoned among the irregulars. Any one who chooses is at liberty to undertake the practice of medicine, but in the penal code of laws established by the present dynasty, malpractice is severely punished. The law is as follows :—

"An unskillful practitioner of medicine who gives drugs or performs acupuncture contrary to the established rules and practice, and thereby, though without any design to injure, kills the patient, shall be allowed to redeem himself from the punishment of homicide, but shall be obliged to quit his profession forever; if it shall appear, however, that he intentionally deviates from the established rules and practice, and aggravates the disease in order to extort more money for its cure, and the patient dies, the money shall be considered as stolen, and the medical practitioner shall be decapitated."

This law, like many others in more civilized countries, is seldom executed. The Chinese are entirely destitute of any correct knowledge of the structure of the human system and of the functions of its organs. There is some reason to suppose that, in ancient times, they possessed greater knowledge of anatomy than at present, but if such was the fact, it has been lost for many centuries.

In their medical books are diagrams and descriptions which exhibit the crude and erroneous notions entertained as to the structure of the human body and the relations of its various parts. It is a matter of astonishment that the comparative anatomy seen in the slaughter-house did not lead them a little nearer the truth; but our wonder at their ignorance will not be so great when we remember that dissection has never been practiced.

The following are some of the most absurd notions taught in their books on this subject: The larynx goes through the lungs to the heart; there are three tubes communicating from the heart to the spleen, liver, and kidneys; the spleen lies between the stomach and diaphragm, the food passing from it into the stomach, and thence by the pylorus into the large intestine.

They believe that the small intestines are connected with the heart, and the urine passes through them into the bladder, separating from the fæces at the caput coli; that the large intestines are connected with the lungs, and lie in the loins, having sixteen convolutions; that the skull, pelvis, arm, and leg, are each single bones. The soul is considered to reside in the liver, and courage in the gall-bladder. Many other instances in which truth and error are mingled, might be mentioned. But while the knowledge of internal parts is limited and imperfect, great attention has been paid to the surface of the body. Every square inch has its appropriate name, the location and relations of which, anatomically and therapeutically, form an important part of the physician's study.

The Chinese possessed a knowledge of the circulation of the blood in very ancient times. It seems, indeed, to have been received as an undisputed fact from time immemorial. Du Halde, a learned Catholic missionary and author of a History of China, published more than a century ago, says they seem to have known it since about 400 years after the deluge. In an ancient treatise on the pulse the circulation is spoken of as a thing about which there is no doubt, and the beating of the pulse is explained by attributing it to the motion of the blood in the vessels. The author reasons thus: "Everything that gives motion, thrusts forward some movable body, and everything that is moved, either gives place readily or makes resistance. Thus, as the blood and spirits are in continual motion, they strike against the vessels in which they are contained, and there must necessarily arise a beating of the pulse."

This extract is taken from a book at least 2000 years old, and others of a similar import might be made from both ancient and modern medical writings. There can be no doubt, therefore, that Chinese physicians have for ages been familiar with a fact, the discovery of which, in Europe, has given immortality to the name of Harvey. We are less surprised at this when we reflect that the art of printing, the mariner's compass, and the manufacture of gunpowder were known to the same people several centuries before they were discovered in Europe.

But while the fact of the circulation has been so long known to Chinese physicians, the most incorrect notions have been, and continue to be, entertained as to the course of the blood and the means by which it permeates every part of the body. It could not be otherwise where dissection was never practiced. The superstitious fears which enslave the minds of all idolaters have ever prevented the examination of man's frame-work after the spirit had taken its flight, and without this its wonderful construction would ever remain a mystery.

It is doubtful if the Chinese make any distinction between veins and arteries, but these are the tubes in which they consider the fluids to move. The action of the heart as a moving power is not clearly understood. In diagrams which illustrate the circulation, several tubes are seen to originate in each of the extremities, and the head, and passing to the thorax, some of them unite together, but they are not made to terminate in any central organ. The rapidity of the blood's movement is underrated, but they are about as near the truth on this point as were our own physiologists until very recently. They estimated that the blood makes two circuits every hour.

One of the most interesting facts connected with the history of Chinese medicine, is the extraordinary attention their physicians have given to the study of the pulse. A most wonderful and complicated system of examining the pulse has been developed and practiced for many centuries. By feeling the pulse alone they profess to tell the seat of a disease, its nature, and result. They profess to tell, also, the cause of a disease, its intensity, and, in fatal cases, how long it will be before death will take place. They profess to decide from the pulse if a woman is pregnant, to tell the stage of her pregnancy, and the sex of the fœtus.

The details of this theory and its unbounded pretensions may incline us to pronounce it all fanciful speculation, and such, no doubt, most of it is; but it cannot be doubted that Chinese physicians have acquired, by long experience, a great skill in judging of the ailments of patients by feeling the pulse. The universal confidence placed in this method, by the common people, is sufficient evidence of the fact. They always expect the pulse at both wrists to be examined, and if the foreign physician neglects this he loses one means of inspiring confidence.

This system dates back to a very early period. The treatise on the pulse already mentioned, is the most ancient, and is considered one of the best works on the subject extant. The author, *Wong Chau Ho,* lived under the *Tsin* dynasty, several centuries before Christ. He acknowledges his work to be a compilation, and divides the books he consulted into ancient and modern.

There are three points in each wrist where the pulse is to be felt, and at each of these points twenty-four varieties are noted, thus making in all 144 different kinds of pulse which the educated physician is expected nicely and accurately to distinguish.

To understand how it is Chinese physicians judge of disease by the pulse, it will be necessary to explain briefly their theories with reference to the sympathetic relations of the various parts of the body and the changes wrought in them by morbific causes.

They lay down two natural principles of life, viz., vital heat and radical moisture, of which the blood and the spirits are the vehicles. In all departments of philosophy they recognize two principles which they call the "*dual powers,*" or the male and female principles of nature, the action and reaction of which give rise to all the phenomena observed in organic and inorganic nature, and it is these two principles which constitute the vital heat and radical moisture.

The body is divided into right and left parts, each of which has an eye, shoulder, arm, hand, leg, and foot. A second division is transverse, into high, middle, and lower parts; and a third into viscera and intestines. The radical moisture is lodged in the six viscera, the heart, spleen, and kidney, on the left; and the lung, liver, and kidney, on the right.

The vital heat resides in the six intestines, viz., the small intestines, gall-bladder, and ureter, on the left; and the large intestine. stomach, and ureter on the right.

It is from these organs that the heat and moisture are distributed by the blood and spirits over the whole body. This is accomplished by means of twelve canals, each one of which takes its origin from one of the twelve seats of heat and moisture.

A very close sympathy is believed to exist between the various internal organs, and each of these is considered to have an intimate relation to one of the five natural elements, earth, air, metals, water, and fire. Thus fire rules the heart; air, the liver and gall-bladder; water, the kidney; metals, the lungs; and earth, the spleen and stomach. The agreement or disagreement of these elements with the various organs deranges the circulation of heat and moisture, and gives rise to alterations and disease.

With this brief sketch of their theory of vitality and of the causes of disease, we will consider how it is they pretend to discover the seat and nature of disorders from the pulse. Each of the three points at the wrist where the pulse is felt is associated with some o l the internal organs, and they believe that all the alterations of every part are manifested by corresponding changes in the appropriate pulse. In short, every internal part has its pulse, of which that at the wrist is the echo or representative.

To show how exceedingly complicated is this system of diagnosis and prognosis, it is only necessary to examine a list of the twenty-four varieties of pulse which are to be distinguished from each other at six different points (three in each wrist.) They are as follows:—

1. A floating pulse.
2. A deep pulse.
3. A slow pulse.
4. A quick pulse.
5. A slippery pulse.
6. A rough pulse.
7. A substantial pulse.
8. An empty pulse.
9. A vibrating pulse.
10. A failing pulse.
11. A rapid pulse.
12. A moderate pulse.
13. A large and broad pulse.
14. A small and minute pulse.
15. A long or protracted pulse.
16. A short pulse.
17. An impeded pulse.
18. A sudden or throbbing pulse.
19. A hidden pulse.
20. A moving pulse.
21. A strong pulse.
22. A weak pulse.
23. A hard pulse.
24. A contracted pulse.

It must be remembered that our language does not contain terms to express the nice distinctions which the corresponding terms convey to the Chinese physician.

To each of these twenty-four varieties of pulse are attached indications for each of the six points where it is felt, and it is from these that the scientific physician judges of the condition of internal organs. As an illustration take the seventeenth variety, which is the "impeded pulse."

The "impeded pulse" in the left wrist,
At 1st point, indicates sudden death.
" 2d " " weakness and perspiration.
" 3d " " exhaustion of the fluids.

The "impeded pulse" in the right wrist,
At 1st point, indicates failing of blood in veins.
" 2d " " water in the stomach.
" 3d " " life endangered.

In treatises on the pulse the most careful directions are given to enable one to arrive at a correct result from the examination of the varieties of the pulse as above explained. Practitioners are also minutely informed as to the modifications of each variety arising from age, sex, habits of life, season of the year, etc. etc. The resemblances and differences of the various kinds of pulse are in like manner carefully pointed out, and physicians are instructed not to confound them, to the great injury of the patient.

An ancient author manifests a commendable zeal for the reputation of his art, and discourses on the subject thus:—"It is necessary to apply one's self diligently to understand the properties of the pulse and to draw the proper consequences, after which, with a sufficient knowledge of drugs, the cure of disease may be undertaken."

Seven cautions are given to a physician about to feel the pulse:—

"1st. He must be in a calm disposition of mind.

"2d. He must be as attentive as possible, laying aside even the smallest disorder or absence of the mind.

"3d. With respect to his body, he should be in a state of tranquillity, and his respiration ought to be free and regular.

"4th. He should, after he has laid his finger softly and touched lightly the skin at the proper places, examine that which regards the six seats of vital heat, viz., the small intestines, gall-bladder, large intestines, etc.

"5th. This done, let him lay on his fingers more hard, moderately pressing the flesh, to examine how the pulse is which is called the pulse of the stomach.

"6th. Then let him press so hard as to feel the bone, and let him examine what relates to the seats of radical moisture, or the heart, liver, stomach, lungs, and kidneys.

"7th. Let him examine the quickness and slowness of the pulse, to see if the number of beats is more or less than it ought to be in the space of one respiration."

It will readily be perceived from the above that this system of examining the pulse is exceedingly complicated, and one which would require long and persevering study to master.

Whatever may be thought of the theories upon which it is founded, we must give the ancient Chinese physicians the credit of being most persevering observers, and although we may reject both their theories and the indications pointed out in their system, yet we can readily conceive how it is possible for one long accustomed to this mode of observation to become very skillful in pointing out the phenomena which are developed in the course of diseased action.

Barrow, in his Travels in China, relates that he was once attacked with cholera morbus, from eating unripe fruit. The Governor of the city, on being applied to for some opium and rhubarb, sent, instead, one of his physicians. "With a countenance as grave and a solemnity as settled as was ever exhibited in a consultation over a doubtful case in London or Edinburgh, he fixed his eyes on the ceiling, while he held my hand, beginning at the wrist and proceeding toward the bend of the elbow, pressing sometimes hard with one finger, and then lightly with another, as if he were running over the keys of a harpsichord. This performance continued for about ten minutes, when he pronounced my complaint to have arisen from eating something that disagreed with my stomach."

Having reviewed thus briefly the theories entertained by our Celestial brethren, let us inquire what is the actual state of the practice of medicine among them. On this subject much remains for us to learn, and our information must continue to be limited and indefinite until foreign physicians, thoroughly conversant with the language, shall come into closer contact with native practitioners, and thus enjoy better opportunities of witnessing the methods of treatment they pursue, and of noting the results.

It is difficult to say whether their practice is influenced more by prevailing theories or by knowledge which has been gained from long experience. Both produce their effects on the mind of the practitioner, and the consequence is that we find a strange mixture of inert and powerful medicines applied in rational and fanciful methods for the removal of disease.

In the treatment of disorders caused by cold or attended with chills, he ating andstimulating medicines are employed, and in febrile affections, medicines supposed to possess refrigerating and sedative properties are used. While they possess medicines having positive virtue, yet there are instances where they rival our homœpathic friends in the exercise of faith. In the winter of 1856 the thermometer fell below the freezing point, (something very uncommon,) one night, at Canton. In the morning an unusual activity was noticed in the streets, and it was found to be caused by people hurrying to the shops to sell ice they had gathered, which was to be bottled for use as a cooling medicine during the summer.

Cathartics, emetics, counter-irritants, and caustics are extensively employed, according to directions which are very minutely detailed in their books. The moxa is also much used, and another favorite and effectual method of counter-irritation is to scrape the skin with a copper coin, or to pinch it many times between two of the coins, until it becomes of a deep livid hue.

Incantations, charms, amulets, astrology, lucky and unlucky days, have an important part to play in most cases of disease. Idols must often be consulted, to decide what doctor shall be called, and various cerémonies must be performed to drive away the evil spirit which is supposed to have taken possession of the sick person. These ceremonies are not of a character to be congenial to the nerves of one prostrated with disease, for an important part of them consists in furiously beating a gong and exploding fire-crackers by the hundred. Another method, more innocent, and possessing a virtue to be ranked with a course of Hahnemann's globules, consists in writing, with red ink, the word which means to dissipate or scatter on the skin over a part where a pain is seated.

The medicines which are used are derived from all departments of nature. In a standard work on Materia Medica and Therapeutics, four hundred and forty-two articles are enumerated and described. They are classified according to their derivation from the animal, vegetable, or mineral kingdoms. Three hundred and fourteen are from the vegetable, fifty from the mineral, and seventy-eight from the animal kingdom.

Many of the articles they use are officinal with us, but the Chinese pharmacopœia stands alone in recognizing the following, which are given as specimens of what are peculiar to themselves:—paws of the bear and monkey, tigers' bones, dried crabs and lizards, dried snake-skins, liver, heart, and marrow of the horse and mule, various parts of the hog and dog, placenta of several animals, dried silk-worms, bear's gall, elephant's skin, etc

The Chinese have long been familiar with the powers of arsenic as an antiperiodic, and they treat syphilis with a preparation made by subliming together mercury and arsenic.

In the compounding of medicines, the utmost attention is directed to be given to several points. The following extract will give the views of writers on this subject:—"Medicines are distinguished into seven sorts; there are simples, which are not joined to any other, and there are compound. Among the latter are those which ought not to be separated from each other, but require to be always together. There are some which lend each other mutual assistance, while between others there is a great antipathy. Great care must be taken in mixing these sorts of medicines. The best way will be to make use of such remedies as cannot exist without each other, and of those which lend mutual assistance. But take great care in using those which have an antipathy among themselves, and which are of a contrary nature. Those that have a malignant or venomous quality may be used, provided they are joined with such as have virtue to subdue this malignity."

Their knowledge of the properties of particular articles seems to be limited, owing, no doubt, to the practice of combining together a great number of ingredients in a single preparation, so that the proper effects of any one of them cannot be known.

Secret remedies are prepared and sold in great abundance, as patent medicines are in the United States. Placards and advertisements are put up at the corners of the streets and in public places, announcing in unmeasured terms the infallible virtues of the nostrum. Jayne, Brandreth, Holloway, and their associates, might learn a profitable lesson if they would study the vocabulary of Chinese quacks. A common mode of winding up an advertisement is to assure the happy consumer of the never-failing pills or powders that "eternally the disease will not return." Some of these nostrums are sold at enormously high prices. Certain pills, for instance, are worth one dollar each. When a high price is paid, the sufferer feels a greater assurance that he is taking a medicine unadulterated with inferior or worthless ingredients.

Considering the great multitude of physicians, and the numerous remedial agents they possess, it must be that centuries of experience have taught them many useful and effectual modes of treating disease. The high estimation in which physicians are held and the great quantity of medicines consumed, are evidences that such must be the case. The proverb says, "A good physician is a public benefactor;" and an eminent physician is called the "*arm of his country.*" The trade in medicines is one of the most important and profitable branches of business. In the large cities, streets are taken up with drug-stores neatly arranged with drawers and jars labeled in the most approved manner. A very noticeable point of difference from our own drug-shops is the absence of glass bottles. Porcelain jars and demijohns take their place. The study of medicinal agents used by the Chinese, and their application of them to the cure of disease, offer very wide fields of investigation to those who have the opportunity to cultivate them.

It is a very curious fact that the practice of surgery is almost entirely unknown to the Chinese. It seems impossible to explain how this could be in a nation where physicians have always been so numerous and so honorable, and where the experience of centuries has been handed down from generation to generation in learned treatises. Whatever may be the reason, it is, nevertheless, a fact, that in all the Empire of China there is not a native surgeon (except a few educated by foreigners,) capable of performing the simplest operation requiring the use of the knife. They possess no surgical instruments. The treatment of fractures and dislocations is not understood. Teeth cannot be extracted until they become loose enough to be removed with the fingers. All diseases and injuries requiring surgical interference, are beyond the reach of the whole native faculty. Foreign physicians who have cured cataract, removed tumors, performed lithotomy, and amputated limbs, are compared to the gods and to physicians of fabulous tradition, and the most extravagant estimate is often placed upon their skill and ability to remove incurable diseases.

The department of midwifery has from time immemorial, and continues to be, exclusively in the hands of females. Native females of respectability lift up their hands in horror and amazement at the idea of having male attendants.

An American lady, long a resident of China and perfectly conversant with the language, says she has often been ashamed to answer their questions upon this point. This is, doubtless, one reason why they look upon foreigners as barbarians, uncivilized, and ignorant of the proprieties of life.

It may, perhaps, be of some interest to the profession to know how lying-in women are treated in the Celestial Empire. The immense number of births which occur annually would seem to render it of the utmost importance that the true principles of the obstetric art should be well understood, and the more so, because the iron rule of custom, by interfering with the growth of the feet, compels the majority of the mothers of China to lead a sedentary life.

It will be seen that the practice of Chinese midwives is the very opposite, in many things, of what we are taught by our distinguished professors in this department. We would, therefore, naturally expect the most disastrous results from what, in America, would be pronounced malpractice. But there is no evidence that those results do follow. On the contrary, so far as I have been able to learn from good sources of information, less of evil is apprehended in their confinements than with us, even when the best obstetrical aid is at hand. Various causes may be assigned for this, one of the principal of which is that they naturally suffer less and have easier deliveries than our own females; but when we consider the luxurious and sedentary habits of many of the people, and the wonderful change which takes place in the most favorable cases of delivery, we will be at a loss to understand how so many escape with so little damage from the ignorance of the professional attendant.

When labor comes on, the midwife is sent for, and the patient sits on a little stool which is placed inside of a low tub. They think it astonishing that any woman can be so uncleanly as to remain in bed. An assistant supports the patient's back and she is always delivered sitting up. It is only in very severe cases that a patient is permitted to lie down. Sometimes they walk about to assist the pains. Medicines are given to expedite delivery, and nearly every woman takes a "hastening pill" at the commencement of labor. The umbilical cord is cut with scissors or a piece of broken porcelain, and is done up in rice flour. The placenta is allowed to come away of itself; but if it is long in doing so, the patient's throat is tickled with a feather and the effort of gagging forces the after-birth away.

After the birth of the child the mother is permitted to lie down a little while, but, as a general thing, she sits up nearly all the time, reclining, perhaps, a little the second day. She is supposed to be able to go about on the third morning, and does so. The poorer classes do not lie down at all, but go about their usual work as soon as the labor is over. Among the wealthy they do not lie in bed, but keep their rooms for one month, being unclean, as they say, and no one is permitted to enter, except those in attendance and very near female relatives. At the end of the month the mother purifies herself, dresses up and comes out to a great feast, on which occasion the child is named, and its head, if a boy, is shaved for the first time by a barber.

Pregnant women continue to eat their usual food up to the time of confinement, but they look forward to this as a time for good eating, and make preparations accordingly. A favorite article is pickled ginger-root. Twenty or thirty pounds of fresh ginger are fried in oil and then boiled in vinegar until they are thoroughly cooked. Very soon after the labor is over, the mother partakes of rice and pickled ginger, with hard boiled eggs, which have also been prepared beforehand. After the second day, if there is no fever, she is allowed chicken, pork, fish, fruit, and whatever she fancies; but everything she takes during the month must be cooked in vinegar and spirits.

As soon as the child is born, it is wiped with soft paper and wrapped up in old clothes of any kind; but it is not washed until the third morning. It is fed with soft-boiled rice immediately after birth, and continues to have two meals of this per day.

No instruments of any kind are used to assist the labor, but to what extent the midwife uses manual interference, I am not informed. It is doubtless a blessing that no one has invented obstetrical instruments for them, while they are so profoundly ignorant of the anatomy of the pelvis and of the mechanism of labor.

The above is an outline of the practice pursued, not in a small community among a few individuals, but throughout the most densely populated empire on the face of the earth. When we contrast it with the rules of action we are accustomed to observe, we are filled with astonishment at the immunity of Chinese females from the calamities incident to the critical period of childbirth. We can only conclude that nature does her work well for them in spite of all difficulties.

### MISSION HOSPITALS.

The first attempt to give the Chinese the benefit of modern medical science, was made by Dr. Alexander Pearson, a surgeon in the service of the East India Company. While residing in Canton and Macao, he undertook the introduction of vaccination among the Chinese. His first efforts were made in 1805, and they were continued for many years, with untiring zeal, amid many difficulties and discouragements, but were at last crowned with success. The prejudices of an ignorant people were overcome, and they were placed in possession of a safeguard against a loathsome pestilence which for centuries had been an annual epidemic.

Dr. Pearson's first report to the National Vaccine establishment was made in 1816, after eleven years of successful labor in his undertaking. He continued to superintend the practice among the natives whom he had instructed, until 1832, or as long as he remained in China. At this time the practice is still kept up in the City of Canton, and many enjoy the exemption secured by vaccination; but for obvious reasons it is not universal, and smallpox annually numbers many victims.

The name of Pearson will long stand on the records of our profession, scarcely less honorable than that of Jenner, a bright example to allure others to like labors of benevolence and charity.

The first hospital for the Chinese was opened by Dr. T. R. Colledge, also a physician in the service of the East India Company. I give in his own words the account of its origin. "In the year 1827, on joining the East India Company's establishment, I determined to devote a large portion of my time, and such medical skill as education and much attention to the duties of my profession had made my own, to the cure of so many poor Chinese sufferers of Macao and its vicinity as came in my way. During that year my own funds supplied the necessary outlay. * * * In 1828, many friends who had witnessed the success of my exertions in the preceding year, came forward to aid in the support of a more regular infirmary which I proposed to establish, and put me in possession of the means to provide for the maintenance of such patients as I found it necessary to keep for some time under my care." In 1832, 4000 Chinese had received medical aid at the hands of Dr. Colledge.

Some of those who had received benefit from treatment, presented written acknowledgments to the physician. The following is a translation of one of the papers presented to Dr. Colledge, and is a specimen of the style in which the Chinese lavish thanks upon the foreign physician:—"While thus extending wide your benevolence, your fame spread over the four seas. I heard thereof, and came, and was happily taken under your care, and not many months passed ere my eyes became bright as the moon and stars when the clouds are rolled away. * * * Now completely cured, and about to return home, I know not when I shall be able to requite your favors and kindness. But, sir, it is the desire of my heart that you may enjoy nobility and emoluments of office, with honor and glory, happiness and felicity that shall daily increase, riches that shall multiply and flourish like the shoots of the bamboo in spring-time, and like that, shall be prolonged ten thousand years. Deeply sensible of your acts of kindness, I have written a few rustic lines which I present with profound respect."

Dr. Colledge's hospital was closed in 1832, on account of the increasing duties of his station, but he continued to manifest great interest in every effort to extend to the Chinese the benefits of scientific medicine and surgery, and his name still stands as President of the Medical Missionary Society.

A Dispensary was opened in 1828 at the City of Canton, by physicians residing there, and much good was done in affording aid and relief to the suffering; but it was not until 1835 that a permanent hospital was established. In that year the Rev. Dr. P. Parker, sent out by the American Board of Foreign Missions, arrived at Canton. Encouraged by the success of Drs. Pearson, Colledge, and others, he rented a building of Howqua, the senior Hong merchant, and commenced receiving patients in November. It was supposed that from so dense a population, one class of cases would afford as many patients as could be treated, and diseases of the eye were selected as the most common and most amenable to treatment. The necessity of the case, however, soon required the admission of other affections, and it became at once a general hospital. At the end of the first year, 2152 patients had been received and treated. The interest of the foreign community in the hospital was manifested by a subscription of $1800 for its support. Dr. Parker continued his labors with increasing success until they were interrupted, in 1840, by the war with England. During the continuance of this, he visited this country and Europe, and much interest was manifested wherever he went, in his statements with reference to medical labors in connection with foreign missions.

The foreign community having witnessed the success which attended the medical labors of Dr. Parker and other physicians, took measures to place on a permanent basis some plan by which these efforts could be sustained. For this purpose, a society was instituted in 1838, called the Medical Missionary Society in China. At the meeting for the formation of this society its object was set forth in the following resolution, viz., "That in order to give a wider extension and a permanency to the efforts that have already been made to spread the benefits of rational medicine and surgery among the Chinese, a society be organized at Canton, under the name of the Medical Missionary Society in China; that the object of this society be to encourage gentlemen of the medical profession to come and practice gratuitously among the Chinese, by affording the usual aid of hospitals, medicine, and attendants, but that the support and remuneration of such medical gentlemen be not at present within its contemplation."

The operations of the Society at Canton and Macao were suspended by the breaking out of hostilities two years after its formtion, but at the re-establishment of peace they were extended to Hong-Kong and some of the new ports which were opened to foreigners by the treaty of Nankin. Under its patronage, hospitals and dispensaries have been established and conducted by physicians connected with various missionary societies in the United States, England, and Continental Europe, and through their labors much has been done to break down that prejudice and arrogant exclusiveness which have ever been the greatest barriers to commercial intercourse and to the introduction of Christianity and science among this wonderful people.

It would be out of place here to give a detailed history of the several hospitals which have been established for the benefit of the Chinese. It will be sufficient to refer to the statistics of the principal ones.

Dr. Benjamin Hobson has been in China since 1839, under the patronage of the London Missionary Society. He has had charge of hospitals, first in Macao and Hong-Kong, and afterwards in Canton. Up to the time of the recent hostilities, not less than 150,000 patients had been treated by him at these places.

At Shanghai a hospital was opened in 1844, by Dr. Lockhart, formerly of Liverpool, who has conducted it with great success up to the present time. I have the statistics of only six years, but during this period 61,201 patients are reported.

The Ophthalmic Hospital of Canton, as already mentioned, was opened in 1835, by Rev. Dr. Parker, who conducted it for eighteen years, during which time the number of patients treated was 53,062. In 1855 the hospital passed into my hands. During two years the number of patients daily received there and at a dispensary, which I conducted at the same time, was from 100 to 170. Both these places were destroyed at the beginning of the recent war with England.

Hospitals and dispensaries have been conducted at Amoy, Fuchau, and Ningpo, on a smaller scale, but with very beneficial results.

The physicians who have had charge of these hospitals have all been sent out by missionary societies in Europe and America, and at all of them the patients have received religious instruction by lectures and the distribution of tracts and portions of the Sacred Scriptures. It has been a primary object in the gratuitous practice of the healing art, to prepare the way for the overthrow of idolatry and superstition, and the introduction of Christianity with all its attendant blessings.

In addition to the appropriate labors of hospital practice, several native young men have received instruction in the principles and practice of our art. One of these, educated by Dr. Parker, and connected with the Ophthalmic Hospital until its recent destruction, has attained to a very respectable standing as a surgeon. He has operated for cataract more than a thousand times, has exsected a great many tumors of various descriptions, and he only needs an acquaintance with practical anatomy to qualify him for the more delicate and dangerous operations. It is to be hoped that the time is not far distant when medical schools will be established which will afford to the young men of the Celestial Empire all the advantages which are now so abundant in Europe and America.

In concluding this imperfect sketch of native and foreign medicine in China, I desire to call the attention of the younger members of our noble profession to the wide field which invites them beyond the borders of their native land. There are some, no doubt, who have chosen the profession of medicine, not as a mercenary trade, but because it affords them so many opportunities for exercising the virtues of benevolence and charity. There may be some who value life and professional attainments only in proportion to the good they are able to do in the use of them. Upon such I would impress the duty of considering the wants of the human family in the Eastern world. There, ignorance, superstition, and idolatry reign supreme, shutting out the benefits which Christianity and modern science have conferred upon the nations of the West.

We congratulate ourselves, and justly, on the wonderful improvements which have recently been made in all departments of medical science. Common philanthropy should inspire us with a desire to extend to all our race that knowledge which enables us to confer so many benefits upon our friends and neighbors. A work which humanity demands of us is the transfer of our knowledge into the Chinese language. It would be worth the labor of a lifetime to any man to give to the physicians of three hundred millions of people such a book as *Wilson's Anatomy.* Who can estimate the good that would result from translations of standard works in each department? A beginning has been made in this work of philanthropy, and the honor of it belongs to Dr. Benjamin Hobson, who has published an Epitome of Anatomy and Physiology, in the Chinese language.

Those who have the light of truth must go forth and dispel this darkness which hovers over the Eastern world. None possess so many advantages for taking the lead in this glorious work as the ardent, pious, and self-denying physician. His skill gains for him access among barbarians, where others cannot go. His disinterested benevolence gives him power and influence, which no others can attain. His daily ministrations to the poor, the lame, the halt, and the blind, are convincing proofs to the most ignorant and prejudiced that no sinister motives influenced his conduct. With such elements of power for good, and with such boundless demands for the exercise of them, our profession will be false to its own honor, false to humanity, and deaf to the wants of suffering millions, if there are none found to go forth and exercise their art where untold benefits will follow their footsteps.

